# Supplementary material for: Research of Liquid-Crystal Materials for a High-Performance FFS-TFT Display
Source: Molecules. 2023 Jan 11;28(2):754. doi: 10.3390/molecules28020754 (PMC9863654; doi:10.3390/molecules28020754)
Supplement: Supplementary file 1 [file molecules-28-00754-s001.zip › molecules-2133411-supplementary.pdf]

# Support Information

# Research of Liquid-Crystal Materials for a High-Performance FFS-TFT Display

**Haiguang Chen [1,2,3], Youran Liu [2,3], Maoxian Chen [2,3], Tianmeng Jiang [2,3], Lanying Zhang [4], Zhou Yang [1,*] and Huai Yang [4,*]**

[1] School of Materials Science and Engineering, University of Science and Technology Beijing, Beijing 100083, China
[2] Beijing Bayi Space Liquid Crystal Material Technology Company, Yangfang 102200, China
[3] Beijing Key Laboratory of Liquid Crystal Materials Analysis and Application Technology, Beijing 102502, China
[4] School of Materials Science and Engineering, Peking University, Beijing 100871, China
* Correspondence: yangz@ustb.edu.cn (Z.Y.); yanghuai@pku.edu.cn (H.Y.); Tel.: +86-01062333759 (Z.Y.); +86-010-62766919 (H.Y.)

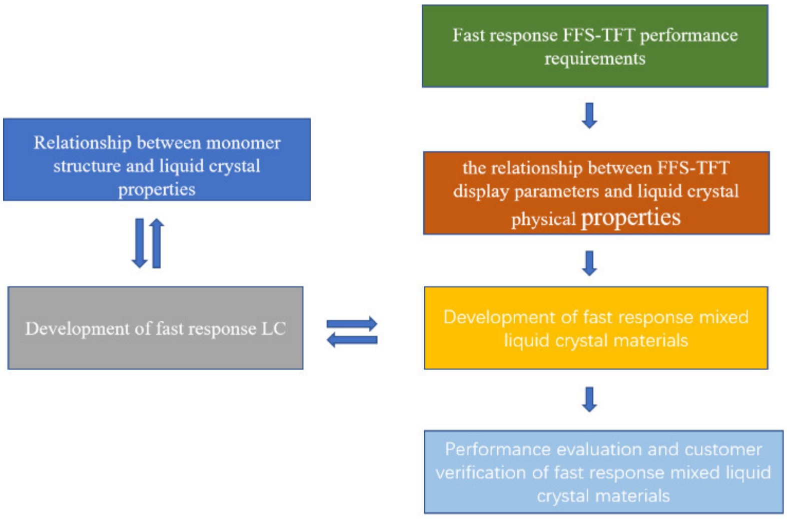


**Figure S1.** Technology Roadmap.

**Table S1.** Spec of Quality

| Liquid Crystal Property | | Spec |
|---|---|---|
| Specific Resistivity [25℃] | ϱ[Ω·cm] | ≥1×$10^{13}$ |
| Specific Resistivity [25℃] After Heat 120℃，2h | ϱ[Ω·cm] | ≥1×$10^{12}$ |
| Resistivity [25℃] After UV 10000mW/$cm^2$ | ϱ[Ω·cm] | ≥1×$10^{12}$ |
| VHR [1V，2s，60℃] | (%) | ≥98.5% |
| VHR [1V，2s，60℃] After UV 10000mW/$cm^2$ | (%) | ≥98% |
| Ion Density [1V，0.01Hz，60℃] | PC | ≤50 |
| Ion Density [1V，0.01Hz，60℃] After UV 10000mW/$cm^2$ | PC | ≤100 |
| Metallic ion | PPb | ≤20 |

**Table S2.** Relationship between Structures and Performances of Liquid Crystal.

| Physical parameters | Adjustment | Monomer structure |
|---|---|---|
| $\Delta\varepsilon$ | ↑ | Dioxane, Linking group |
| | ↓ | Nonpolar terminal |
| $\Delta n$ | ↑ | Increase number of rings,  Phenyl |
| | ↓ | Cyclohexane, Dioxane |
| $T_{S\text{-}N}$ | ↑ | Linking group,  F substitution |
| | ↓ | Polycyclic |
| Cp | ↑ | Cyclohexane, Polycyclic,Long carbon chain |
| | ↓ | Phenyl |
| $\gamma 1$ | ↑ | Cyclohexane, Long carbon chain |
| | ↓ | Phenyl, Dioxane, Alkenyl linking group |
| K11 | ↑ | Cyclohexane，Polycyclic |
| | ↓ | Phenyl |
| K22 | ↑ | Cyclohexane，Polycyclic |
| | ↓ | Phenyl |
| K33 | ↑ | Cyclohexane，Polycyclic，Alkenyl |
| | ↓ | Phenyl |

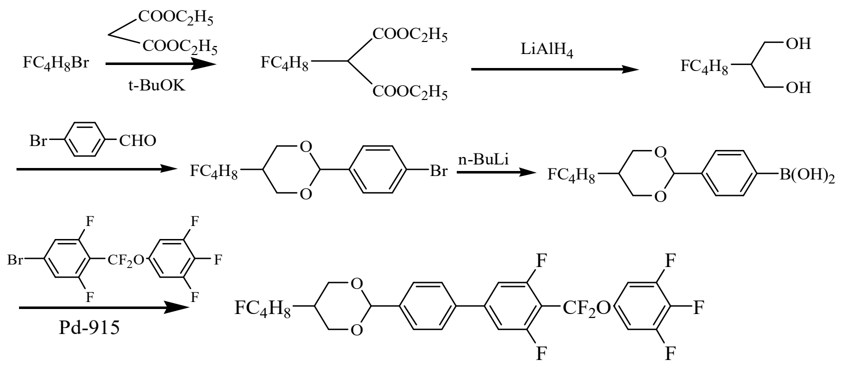


**Figure S2.** The synthesized route of the TFT liquid crystal compound.

**Table S3.** The contents of some metallic Ions

| | BHR95300 | | |
|---|---|---|---|
| Na | 0 | Ni | 0 |
| Mg | 0.02 | Cu | 0.69 |
| Al | 0.02 | Zn | 0 |
| K | 0.13 | Sr | 0 |
| Mn | 0 | Pd | 0 |
| Fe | 0 | Sn | 0.09 |
| Co | 0 | Total | 0.95 |
